# A Phylogenetic and Taxonomic Study on *Phellodon* (Bankeraceae, Thelephorales) from China

**DOI:** 10.3390/jof8050429

**Published:** 2022-04-22

**Authors:** Chang-Ge Song, Yuan-Yuan Chen, Shun Liu, Tai-Min Xu, Xiao-Lan He, Di Wang, Bao-Kai Cui

**Affiliations:** 1 Institute of Microbiology, School of Ecology and Nature Conservation, Beijing Forestry University, Beijing 100083, China; changgesong@126.com (C.-G.S.); liushun2017@bjfu.edu.cn (S.L.); fungitaiminx@bjfu.edu.cn (T.-M.X.); 2 College of Forestry, Henan Agricultural University, Zhengzhou 450002, China; cyuan091@163.com; 3 Sichuan Institute of Edible Fungi, Sichuan Academy of Agricultural Sciences, Chengdu 610066, China; xiaolanhe1121@aliyun.com (X.-L.H.); wang.di19881213@163.com (D.W.)

**Keywords:** ectomycorrhizal fungi, molecular phylogeny, morphology, stipitate hydnoid fungi

## Abstract

In this study, phylogenetic analyses of *Phellodon* from China were carried out based on sequences from the internal transcribed spacer (ITS) regions, the large subunit of nuclear ribosomal RNA gene (nLSU), the small subunit of nuclear ribosomal RNA gene (nSSU), the largest subunit of RNA polymerase II (RPB1), and the second largest subunit of RNA polymerase II (RPB2), combined with morphological characters of the collected specimens in China. The fruiting bodies of the specimens were used to observe their characteristics, and three new species of *Phellodon* are discovered. *Phellodon crassipileatus* is characterized by its pale brown to dark brown pileal surface, tomentose pileal margin, white spines, and the presence of clamp connections in generative hyphae of pileal surface, context, and stipe. *Phellodon griseofuscus* is characterized by its dark brown to black pileal surface, white to pale brown pileal margin, the presence of both simple septa and clamp connections in generative hyphae of spines, and moderately long basidia. *Phellodon perchocolatus* is characterized by its woody and broad pileus, brown to greyish brown pileal surface when fresh, tomentose pileal margin when young, which becomes glabrous with age, and the presence of both simple septa and clamp connections in the generative hyphae of the spines. This is the first time both single and multi-genes analysis is used in such a phylogenetic and taxonomic study on *Phellodon*, which can provide the basis for the phylogenetic study of the genus.

## 1. Introduction

*Phellodon* P. Karst. was established by Petter Adolf Karsten and typified by *P. niger* (Fr.) P. Karst [1]. The genus, together with *Hydnellum* P. Karst. and *Sarcodon* Quél. ex P. Karst. were stipitate hydnoids, and they were affiliated to Bankeraceae of Thelephorales. All of the three genera belong to ectomycorrhizal fungi, which are associated with broad-leaved or coniferous trees in forest ecological systems [2,3,4]. Ectomycorrhizal fungi are symbionts of trees in forests, which can reflect the conservation state of forest ecosystems [5]. They can connect plant roots to soil by promoting the decomposition of organic matter in soil and the absorption of organic and inorganic elements by host plants [6]. Therefore, they are of great significance to the growth of plants and the material circulation of ecosystems. During the second half of the 20th century, the numbers of most species of stipitate hydnoid fungi have declined [7], and many species have been included in national red lists [8]. This is most likely ascribed to habitat loss due to forestry operations, such as massive logging, the disappearance of old *Picea* forests on calcareous soils, deciduous forest transformed into coniferous forest, direct effects of air pollutants, and forest soil acidification [7,9]. In addition, sulfur and nitrogen depositions and soil acidification also contribute to the decline of stipitate hydnoid fungi [7,9,10]. In recent decades, the number of stipitate hydnoid fungi has dropped significantly, which reflects that we need to pay more attention to protecting them [4]. Meanwhile, discovering new species of stipites hydnoid fungi is also of great significance for helping us to further recognize and protect them.

Macro-morphologically, species of *Phellodon*, *Hydnellum*, and *Sarcodon* are relatively similar in having single to concrescent basidiomata and spines. However, the three genera can be distinguished by the color of their basidiospores. Traditionally, species in *Hydnellum* and *Sarcodon* have brown basidiospores, while species in *Phellodon* have white basidiospores [4]. While in a recent comprehensive study, Larsson et al. [11] suggested that basidiospore size can distinguish the *Hydnellum* and *Sarcodon*, species in *Hydnellum* have basidiospore lengths in the range 4.45−6.95 µm while the corresponding range for *Sarcodon* is 7.4−9 µm. Species in *Phellodon* are characterized by solitary to gregarious or concrescent, stipitate basidiomata, hydnoid hymenophore, and echinulate basidiospores [12], and often occur in forests of Fagaceae and Pinaceae [2,6].

In 1881, Karsten divided the genus *Hydnellum* into two parts: the white toothed and the dark toothed, and the former was named *Phellodon* [13]. Banker [13] revised all of the Hydnaceae found in the continent of North America and its adjacent areas, which included *Hydnellum*, *Phellodon*, and *Sarcodon*, and 10 species of *Phellodon* were described based on morphological features. Species of *Phellodon* were described only based on morphological characteristics in the past few decades, which resulted in the lack of molecular basis for taxonomic studies of the genus [11,13,14,15,16,17,18,19,20,21,22,23,24,25]. Morphological and phylogenetic studies were used to identify the genus in recent years. Parfitt et al. [4] carried out a systematic study of *Hydnellum* and *Phellodon* based on molecular and morphological analyses, which identified the taxonomic status of the known *Phellodon* species from Britain. Ainsworth et al. [26] revealed the cryptic taxa of the genera *Hydnellum* and *Phellodon* based on the combination of molecular and morphological analysis. Moreover, Baird et al. [27] reevaluated the species of stipitate hydnums from the southern United States, and 41 distinct taxa of *Hydnellum*, *Phellodon*, and *Sarcodon* were determined. At the same time, they described 10 species of *Phellodon*. They provided phylogenetic analyses on *Phellodon* based on ITS sequences, which provided a morphological and molecular basis for taxonomic and phylogenetic studies of the genus. Furthermore, *Bankera fuligineoalba* (J.C. Schmidt) Pouzar, the typified species of *Bankera* Coker and Beers, was recombined in *Phellodon* in their study, which suggests that the genus *Bankera* has already been combined into *Phellodon.* In recent years, the genus has been studied in China. Mu et al. [12] described *Phellodon subconfluens* H.S. Yuan and F. Wu in Liaoning Province based on morphological characters and molecular data. Later, Song et al. [28] described four species of *Phellodon, P. atroardesiacus* B.K. Cui and C.G. Song, *P. cinereofuscus* B.K. Cui, and C.G. Song, *P. stramineus* B.K. Cui, and C.G. Song and *P. yunnanensis* B.K. Cui, and C.G. Song, based on morphological characters and ITS sequences data from southwestern China [28].

During the investigations of stipitate hydnoid fungi from China, abundant fruiting bodies were obtained, and three undescribed species of *Phellodon* were discovered. To confirm the affinity of the undescribed species corresponding to *Phellodon*, phylogenetic analyses were carried out based on ITS and ITS + nLSU + nSSU + RPB1 + RPB2 sequences. The new species were described based on the combination of morphological and phylogenetic analysis.

## 2. Materials and Methods

### 2.1. Morphological Studies

Methods of specimen collection and preservation followed the methods of Wang [29]. The specimens used in this study were collected during the annual growing season of macrofungi. At the same time, the specimen information, host trees, ecological habits, location, altitude, collector, date were recorded, and the photos of the fruiting bodies and growth environment were taken. Then, the specimens were dried and bagged in time for preservation. After that, the specimens were registered and deposited at the herbarium of the Institute of Microbiology, Beijing Forestry University (BJFC). Macromorphological descriptions were based on the field notes and measurements of herbarium specimens. Microscopic characteristics, measurements, and drawings were made from slide preparations stained with Cotton Blue and Melzer’s reagent and observed at magnifications up to ×1000 under a light microscope (Nikon Eclipse E 80i microscope, Nikon, Tokyo, Japan) following Liu et al. [30]. Basidiospores were measured from sections cut from the spines. The following abbreviations are used: IKI, Melzer’s reagent; IKI–, neither amyloid nor dextrinoid; KOH, 5% potassium hydroxide; CB, Cotton Blue; CB–, acyanophilous; L, mean spore length (arithmetic average of all spores); W, mean spore width (arithmetic average of all spores); Q, variation in the L/W ratios between the specimens studied; n (a/b), and number of spores (a) measured from given number (b) of specimens. A field Emission Scanning Electron Microscope (FESEM) Hitachi SU-8010 (Hitachi, Ltd., Tokyo, Japan) was used to film the spore’s morphology. Sections were studied at up to 2200 times magnification, according to the method by Sun et al. [31].

### 2.2. Molecular Study and Phylogenetic Analysis

DNA extraction, amplification, and sequencing: the CTAB rapid plant genome extraction kit (Aidlab Bio technologies Co., Ltd., Beijing, China) was used to obtain PCR products from dry specimens, and for polymerase chain reaction (PCR), according to the manufacturer’s instructions with some modifications [32]. The primer pairs ITS5/ITS4, LR0R/LR7, NS1/NS4, AF/Cr, and 5F/7Cr were used to amplify ITS, nLSU, nSSU, RPB1, and RPB2 sequences [28]. The PCR process for ITS was as follows: initial denaturation at 95 °C for 3 min, followed by 35 cycles at 94 °C for 40 s, 56 °C for 45 s and 72 °C for 1 min and a final extension of 72 °C for 10 min. The PCR process for nLSU and nSSU was as follows: initial denaturation at 94 °C for 1 min, followed by 35 cycles at 94 °C for 30 s, 50 °C for 1 min and 72 °C for 90 s and a final extension of 72 °C for 10 min. The PCR process for RPB1 and RPB2 was as follows: initial denaturation at 94 °C for 2 min, 9 cycles at 94 °C for 45 s, 60 °C for 45 s, followed by 36 cycles at 94 °C for 45 s, 53 °C for 1 min, 72 °C for 90 s and a final extension of 72 °C for 10 min. The PCR products were purified and sequenced in Beijing Genomics Institute, China, with the same primers. All newly generated sequences were submitted to GenBank and are listed in (Table 1). Moreover, other sequences in the dataset for phylogenetic analysis were downloaded from GenBank (http://www.ncbi.nlm.nih.gov/genbank/php, accessed on 15 October 2021).

New sequences generated in this study were aligned with additional sequences downloaded from GenBank (Table 1) using ClustalX [33] and manually adjusted in BioEdit [34]. The sequences of *Amaurodon aquicoeruleus* Agerer and *A. viridis* (Alb. and Schwein.) J. Schröt. were used as the outgroups, according to Mu et al. [12].

Maximum parsimony (MP) analysis followed and was applied to the sequence datasets using PAUP* version 4.0b10 [35], and the congruences of the 5-gene (ITS, nLSU, nSSU, RPB1, and RPB2) were evaluated with the incongruence length difference (ILD) test [36]. Gaps in the alignments were treated as missing data. Maxtrees were regular to 5000, branches of zero length were collapsed, and all parsimonious trees were saved. Clade might be assessed using a bootstrap (BS) analysis with 1000 replicates [37]. Descriptive tree statistics tree length (TL), consistency index (CI), retention index (RI), rescaled consistency index (RC), and homoplasy index (HI) were calculated for each maximum parsimonious tree generated.

Maximum likelihood (ML) analysis was conducted with RAxML-HPC252 on Abe through the Cipres Science Gateway (www.phylo.org, accessed on 18 October 2021), which referred to 100 ML searches, and the program estimated all model parameters. The maximum likelihood bootstrap (ML-BS) values were performed with a rapid bootstrapping with 1000 replicates. Phylogenetic trees were viewed using FigTree v1.4.2 (http://tree.bio.ed.ac.uk/software/figtree/, accessed on 18 October 2021).

MrModeltest2.3 [38,39] was used to determine the best-fit evolution model for the combined dataset for Bayesian inference (BI). BI was performed using MrBayes 3.2.6 on Abe through the Cipres Science Gateway (www.phylo.org, accessed on 19 October 2021) with 2 independent runs, beginning from random trees with 4 simultaneous independent Chains, performing 2 million replicates, sampling 1 tree for every 100 generations. The burn-in was set to discard 25% of the trees. The remaining ones were used to construct a majority rule consensus and calculate the Bayesian posterior probabilities (BPP) of the clades.

Branches that received bootstrap support for maximum parsimony (MP), maximum likelihood (ML), and Bayesian posterior probabilities (BPP) greater than or equal to 50% (MP and ML) and 0.95 (BPP) were regarded as prominently supported.

## 3. Results

### 3.1. Phylogenetic Analyses

**Figure 1:**
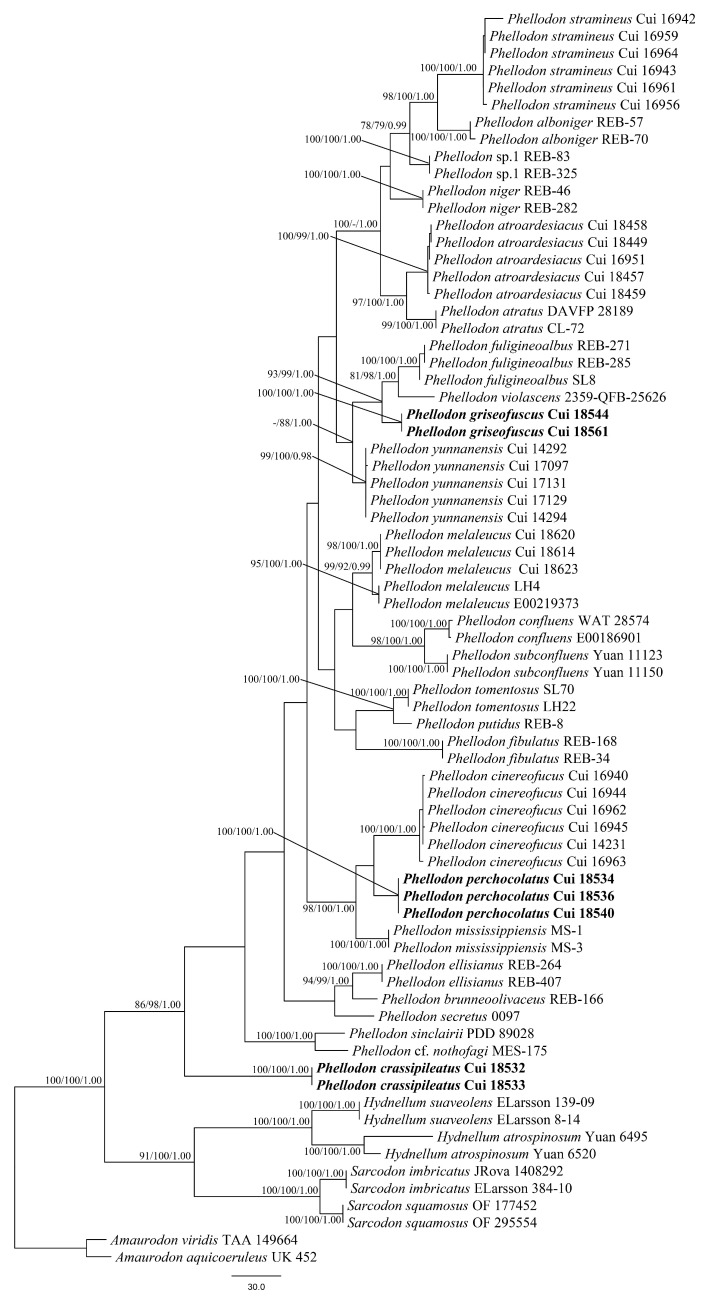
Maximum parsimony (MP) phylogram of the *Phellodon* species based on ITS sequences data. The supported branches are labeled with parsimony bootstrap values higher than 75%, maximum likelihood bootstrap values higher than 75%, and Bayesian posterior probabilities more than 0.95. Bold names = New species.

The dataset of ITS included 73 sequences representing 32 taxa. The ITS dataset had an aligned length of 873 characters, of which 376 characters were constant, 46 were variable and parsimony-uninformative, and 451 were parsimony-informative. Maximum parsimony analysis yielded 516 equally parsimonious trees (TL = 1516, CI = 0.547, RI = 0.839, RC = 0.459, HI = 0.453), and 1 of the maximum parsimonious trees is shown in Figure 1. The best fit model selected for these three partitions of ITS sequences was GTR + G for ITS1, JC for 5.8 s, and HKY + G for ITS2. BI resulted in a similar topology with an average standard deviation of split frequencies = 0.007630 to MP analysis. The MP topology is shown with MP (≥75%), ML (≥75%), and BPP (≥0.95) supported values at the nodes (Figure 1).

In the ITS based phylogenetic tree (Figure 1), the three new species *P. crassipileatus*, *P. griseofuscus*, and *P. perchocolatus* formed distinct well-supported lineages distant from other species of *Phellodon*.

**Figure 2:**
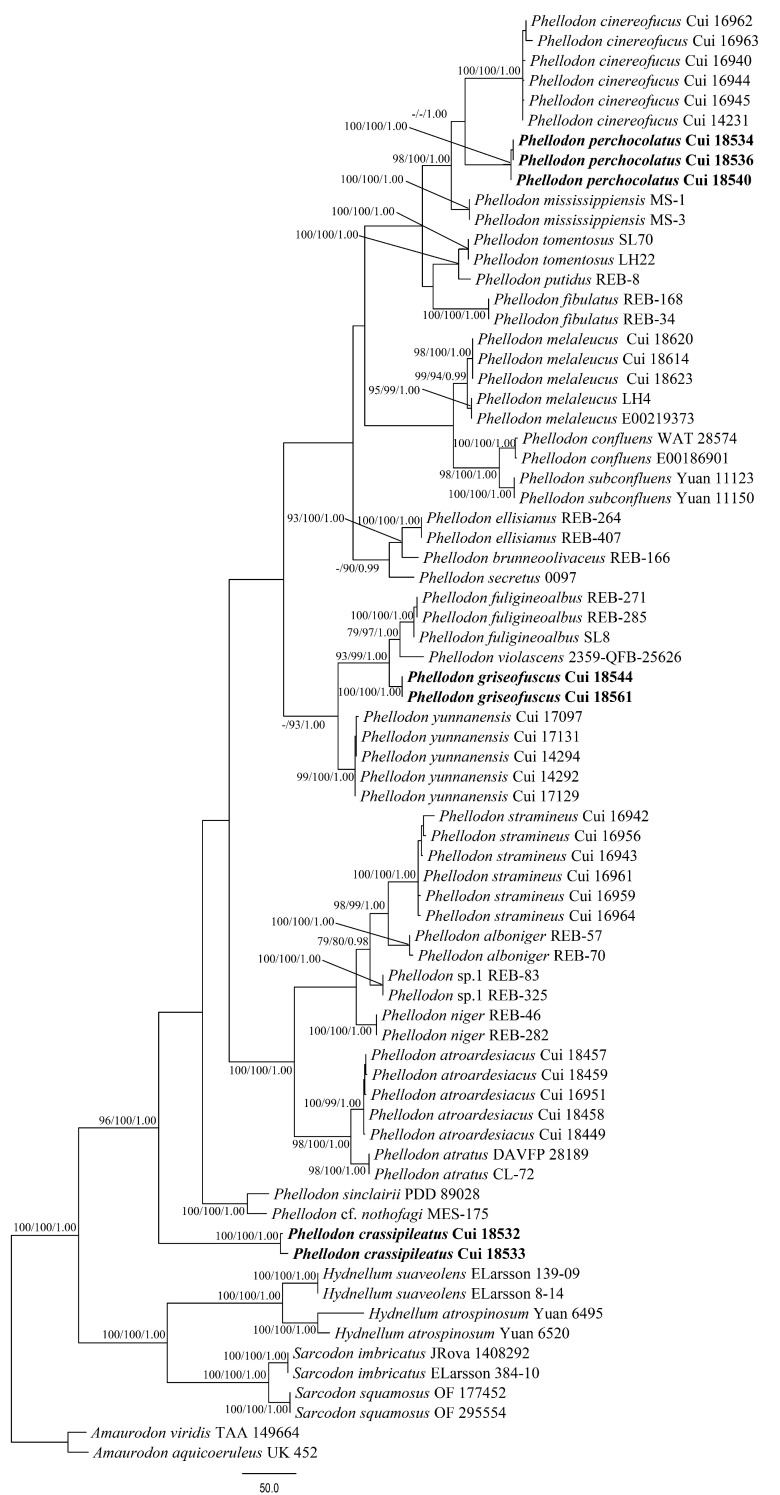
Maximum parsimony (MP) phylogram of the *Phellodon* species based on the combined ITS + nLSU + nSSU + RPB1 + RPB2 sequences data. The supported branches are labeled with parsimony bootstrap values higher than 75%, maximum likelihood bootstrap values higher than 75%, and Bayesian posterior probabilities more than 0.95. Bold names = New species.

The combined ITS + nLSU + nSSU + RPB1 + RPB2 dataset included sequences from 73 fungal samples representing 32 taxa. The combined dataset had an aligned length of 5639 characters including gaps (873 characters for ITS, 1379 characters for nLSU, 1097 characters for nSSU, 1203 characters for RPB1, 1087 characters for RPB2), of which 4599 characters were constant, 192 were variable and parsimony-uninformative, and 848 were parsimony-informative. Maximum parsimony analysis yielded 12 equally parsimonious trees (TL = 2195, CI = 0.643, RI = 0.866, RC = 0.557, HI = 0.357), and 1 of the maximum parsimonious trees is shown in Figure 2. The best fit model selected for the combined ITS + nLSU + nSSU + RPB1 + RPB2 sequence dataset was GTR + I+G with equal frequency of nucleotides. BI resulted in a similar topology with an average standard deviation of split frequencies = 0.008906 to MP analysis. The MP topology is shown with MP (≥75%), ML (≥75%), and BPP (≥0.95) supported values at the nodes (Figure 2).

The ITS + nLSU + nSSU + RPB1 + RPB2 based phylogenetic tree (Figure 2) produced a topology similar to that generated by the ITS based phylogenetic tree, and confirmed the affinities of the three new species within *Phellodon*.

### 3.2. Taxonomy

**Figure 3:**
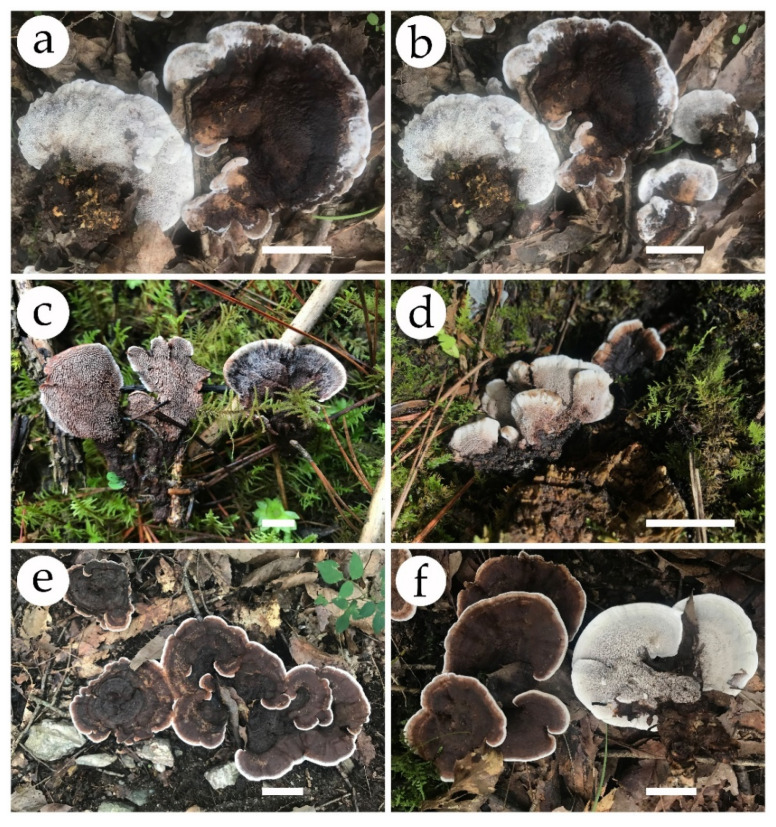
Basidiomata of *Phellodon* species. (**a**,**b**) *P. crassipileatus*, (**c**,**d**) *P. griseofuscus*, and (**e**,**f**) *P. perchocolatus.* Scale bars: 2 cm.

**Figure 4:**
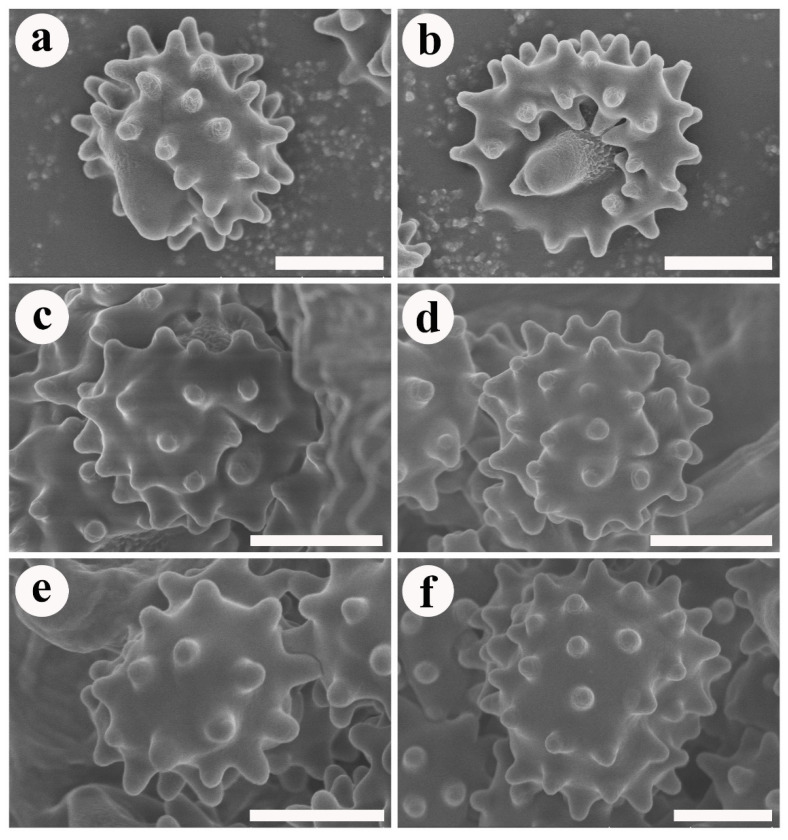
SEM of basidiospores of *Phellodon* species. (**a**,**b**) *P. crassipileatus*, (**c**,**d**) *P. griseofuscus*, and (**e**,**f**). *P. perchocolatus*. Scale bars: 1.5 µm.

**Figure 5:**
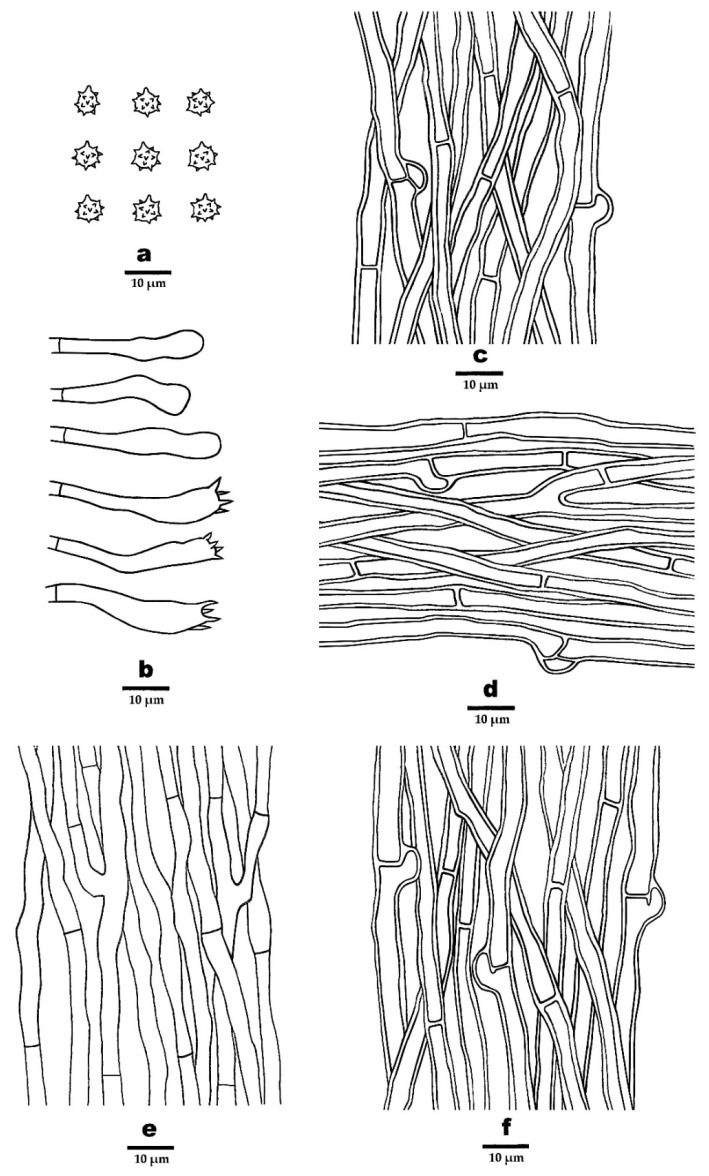
Microscopic structures of *P. crassipileatus* (drawn from the holotype). (**a**) Basidiospores, (**b**) Basidia and basidioles, (**c**). Hyphae from pileal surface, (**d**). Hyphae from context, (**e**). Hyphae from spines, and (**f**) Hyphae from stipe.

***Phellodon crassipileatus*** B.K. Cui and C.G. Song, sp. nov., Figure 3a, Figure 4a,b, and Figure 5.

MycoBank: 843670

**Diagnosis**—This species is characterized by its pale brown to dark brown pileal surface, thick pileus, tomentose pileal margin, white spines, and the presence of clamp connections in generative hyphae of pileal surface, context, and stipe.

**Etymology**—*crassipileatus* (Lat.): refers to the thick pileus.

**Holotype**—CHINA. Sichuan Province, Pingwu County, Bazi, on the ground of forest dominated by trees of *Quercus* sp., alt. 1190 m, 18 September 2020, Cui 18533 (BJFC 035394).

**Fruiting body**—Basidiomata annual, centrally or eccentrically stipitate, solitary or gregarious, with a fenugreek odor when dry. Pileus infundibuliform, up to 6.5 cm in diam, 2 cm thick at the center. Pileal surface pale brown to dark brown when fresh, becoming dark brown upon drying, azonate, tomentose at the margin; pileal margin blunt or irregular, white when fresh, becoming cream upon drying, up to 1.2 cm wide. Spines soft, white when fresh, becoming fragile, cream to clay-buff upon drying, up to 3 mm long. Context vinaceous grey, tough, up to 6 mm thick. Stipe brown to dark brown in the outer layer, fuscous in the inner layer, cylindrical, glabrous, up to 1.5 cm long, 1 cm in diameter.

**Hyphal structure**—Hyphal system monomitic; generative hyphae mostly with simple septa, occasionally with clamp connections; all the hyphae IKI–, CB–; tissues turned to olive green in KOH. Generative hyphae in pileal surface pale brown, thick-walled, rarely branched, mostly with simple septa, occasionally with clamp connections, parallel, 2–6 µm in diameter. Generative hyphae in context clay-buff to pale brown, thick-walled, occasionally branched, mostly with simple septa, occasionally with clamp connections, 2–5 µm in diameter. Generative hyphae in spines clay-buff to pale brown, thin-walled, branched, with simple septa, more or less parallel along the spines, 2–4 µm in diameter. Generative hyphae in stipe clay-buff to brown, thick-walled, rarely branched, mostly bearing simple septa, occasionally with clamp connections, parallel along the stipe, 2–6 µm in diameter.

**Cystidia**—Cystidia and other sterile hyphal elements absent.

**Basidia**—Basidia clavate, bearing four sterigmata and a basal simple septum, 22–45 × 4–7 µm; sterigmata, 1.5–5 µm; basidioles similar to basidia in shape, but slightly smaller.

**Spores**—Basidiospores subglobose to globose, hyaline, thin-walled, echinulate, IKI–, CB–, (3.5–) 4–5 × 4–5 µm, L = 4.67 µm, W = 4.19 µm, Q = 1–1.25 (n = 60/2, without the ornamentation).

**Additional specimen (paratype) examined**—China, Sichuan Province, Pingwu County, Bazi, on the ground of forest dominated by trees of *Quercus* sp., alt. 1190 m, 18 September 2020, Cui 18532 (BJFC 035393).

**Ecological habits**—*P. crassipileatus* was found on the ground of forest dominated by trees of *Quercus* sp., under a humid monsoon-climate in the northern subtropical region.

**Figure 6:**
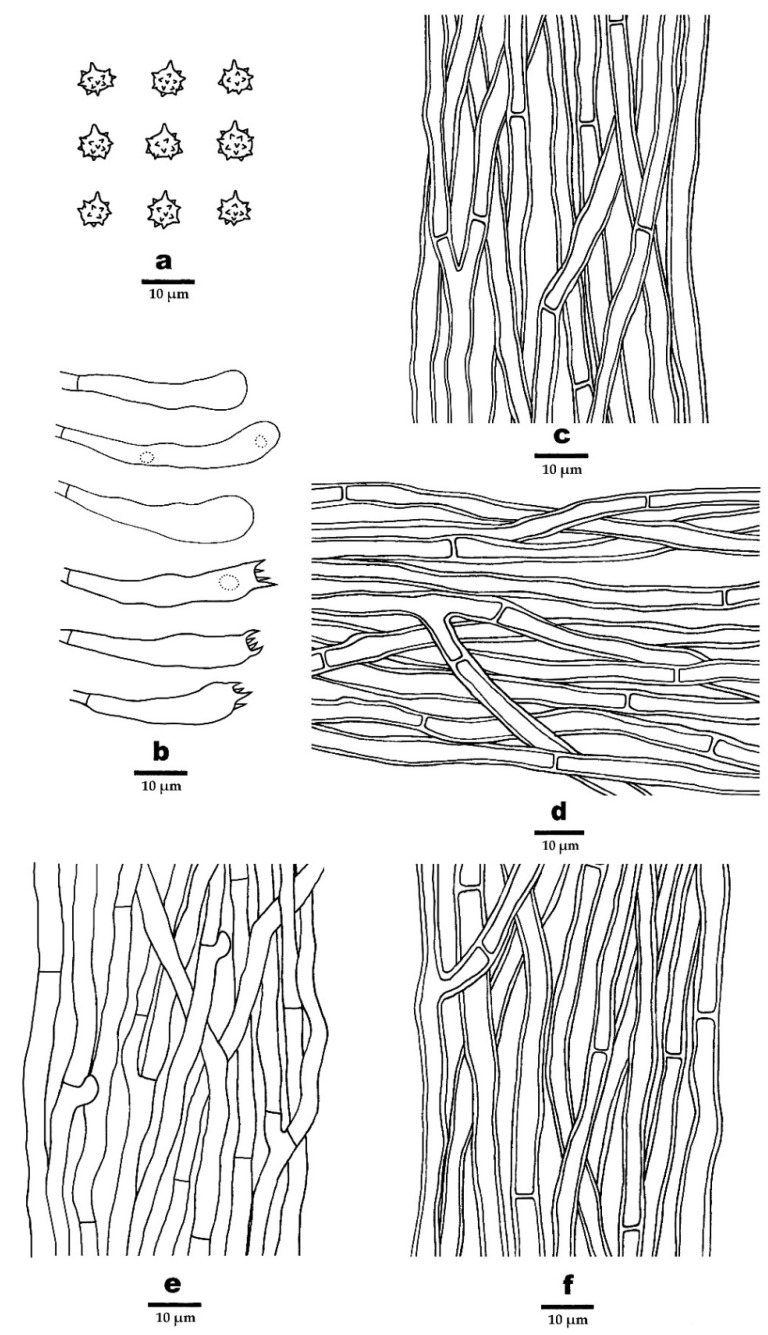
Microscopic structures of *P. griseofuscus* (drawn from the holotype). (**a**) Basidiospores, (**b**) Basidia and basidioles, (**c**) Hyphae from pileal surface, (**d**) Hyphae from context, (**e**) Hyphae from spines, and (**f**) Hyphae from stipe.

***Phellodon griseofuscus*** B.K. Cui and C.G. Song, sp. nov., Figure 3b, Figure 4c,d, and Figure 6.

MycoBank: 843671.

**Diagnosis**—This species is characterized by its dark brown to black pileal surface, white to pale brown pileal margin, short spines, generative hyphae with both simple septa and clamp connections in spines, and moderately long basidia.

**Etymology**—*griseofuscus* (Lat.): refers to the pale brown to dark brown or blackish basidiomata.

**Holotype**—China, Sichuan Province, Jiuzhaigou County, Jiuzhaigou Nature Reserve, on the ground of forest dominated by trees of *Pinus* sp., alt. 2400 m, 20 September 2020, Cui 18561 (BJFC 035422).

**Fruiting body**—Basidiomata annual, centrally or eccentrically stipitate, solitary or gregarious, with strong odor when dry. Pileus infundibuliform, up to 4 cm in diameter, 5 mm thick at the center. Pileal surface pale brown to dark brown or black when fresh and becoming dark grey to mouse-grey upon drying, azonate, fibrillose; margin blunt or irregular, white to pale brown when fresh, vinaceous grey with age, becoming fuscous upon drying, up to 3 mm wide. Spines soft, white when young, brown with age when fresh, becoming fragile, pale mouse-grey upon drying, up to 1 mm long. Context dark grey, tough, up to 2 mm thick. Stipe fuscous in the outer layer, fuscous to black in the inner layer, cylindrical, glabrous, up to 1.5 cm long, 0.6 cm in diameter.

**Hyphal structure**—Hyphal system monomitic; generative hyphae mostly with simple septa, occasionally with clamp connections; all the hyphae IKI–, CB–; tissues turned to olive green in KOH. Generative hyphae in pileal surface greyish brown, thick-walled, rarely branched, with simple septa, parallel, 3–6 µm in diameter. Generative hyphae in context pale brown, thick-walled, occasionally branched, with simple septa, parallel, 3–5 µm in diameter. Generative hyphae in spines clay-buff, thin-walled, branched, mostly with simple septa, occasionally with clamp connections, more or less parallel along the spines, 2–4 µm in diameter. Generative hyphae in stipe greyish brown, slightly thick-walled, rarely branched, bearing simple septa, subparallel along the stipe, 2–6 µm in diameter.

**Cystidia**—Cystidia and other sterile hyphal elements absent.

**Basidia**—Basidia clavate, bearing four sterigmata and a basal simple septum, 22–55 × 5–6 µm; sterigmata, 1.5–5µm; basidioles similar to basidia in shape, but slightly smaller.

**Spores**—Basidiospores subglobose to globose, hyaline, thin-walled, echinulate, IKI–, CB–, 4–5 × 3.5–4.5 µm, L = 4.4 µm, W = 4 µm, Q = 1–1.25 (n = 60/2, without the ornamentation).

**Additional specimen (paratype) examined**—China, Sichuan Province, Jiuzhaigou County, Jiuzhaigou Nature Reserve, on the ground of forest dominated by trees of *Pinus* sp., alt. 2400 m, 19 September 2020, Cui 18544 (BJFC 035405).

**Ecological habits**—*P. griseofuscus* was found on the ground of forest dominated by trees of *Pinus* sp., under the humid climate of the plateau. This species grows in well-watered bryophytes, which are often interspersed with pine needles.

**Figure 7:**
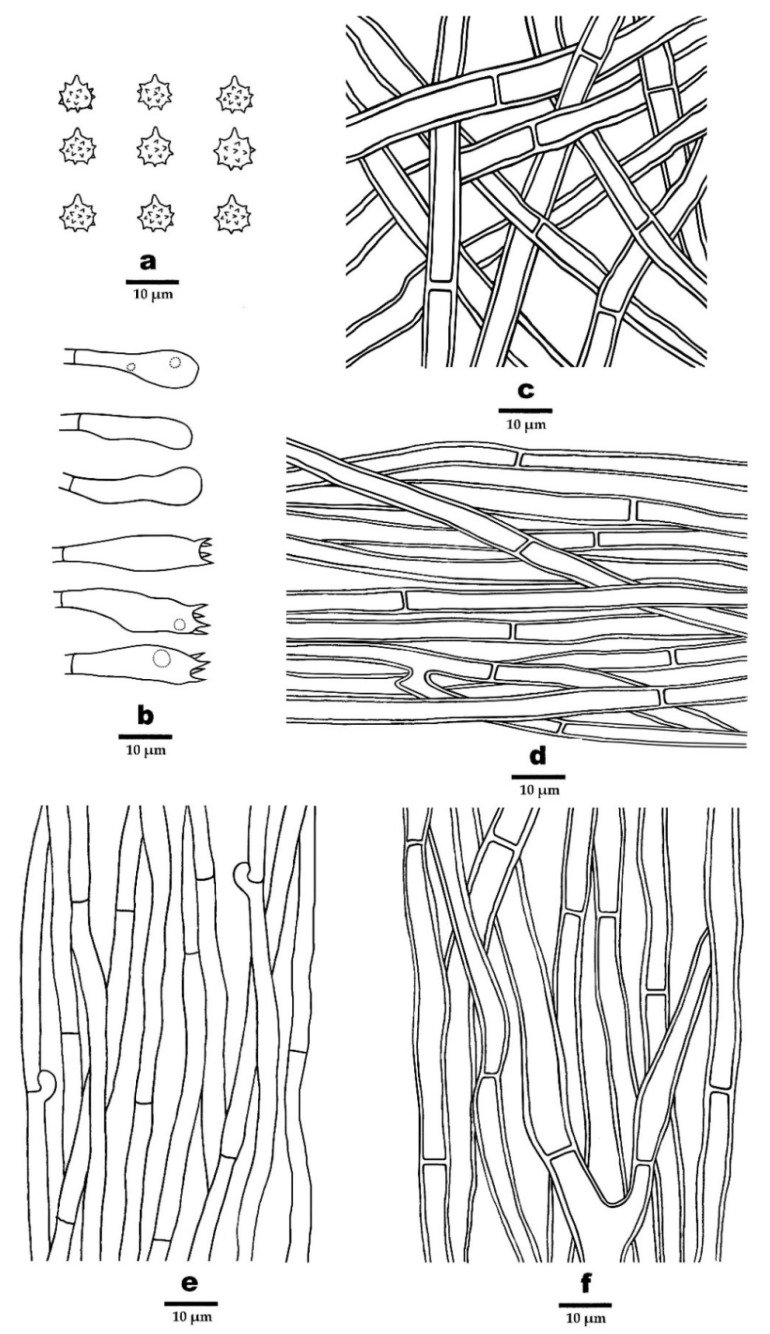
Microscopic structures of *P. perchocolatus* (drawn from the holotype). (**a**) Basidiospores, (**b**) Basidia and basidioles, (**c**). Hyphae from pileal surface, (**d**) Hyphae from context, (**e**) Hyphae from spines, and (**f**) Hyphae from stipe.

***Phellodon perchocolatus*** B.K. Cui and C.G. Song, sp. nov., Figure 3c, Figure 4e,f, and Figure 7.

MycoBank: 843672

**Diagnosis**—This species is characterized by its woody and broad pileus, brown to greyish brown pileal surface when fresh, tomentose pileal margin when young and glabrous after mature, and the presence of both simple septa and clamp connections in generative hyphae of spines.

**Etymology**—*perchocolatus* (Lat.): refers to the brown to greyish brown pileal surface.

**Holotype**—China, Sichuan Province, Pingwu County, Bazi, on the ground of forest dominated by trees of *Quercus* sp., alt. 1190 m, 18 September 2020, Cui 18536 (BJFC 035397).

**Fruiting body**—Basidiomata annual, centrally or eccentrically stipitate, solitary or gregarious, with a fenugreek odor when dry. Pileus infundibuliform, woody, up to 9 cm in diam, 6.5 mm thick at the center. Pileal surface brown to greyish brown when fresh, becoming fuscous to black upon drying, zonate, tomentose when young, glabrous after the mature; margin blunt or irregular, white when fresh, becoming buff upon drying, up to 5 mm wide. Spines soft, white when fresh, and becoming fragile, pinkish buff to olivaceous buff upon drying, up to 3 mm long. Context vinaceous grey to greyish brown, tough, up to 3 mm thick. Stipe dark brown to fuscous in the outer layer, fuscous in the inner layer, cylindrical, glabrous, up to 4.8 cm long, 1.9 cm in diameter.

**Hyphal structure**—Hyphal system monomitic; generative hyphae mostly with simple septa, occasionally with clamp connections; all the hyphae IKI–, CB–; tissues turned to olive-green in KOH. Generative hyphae in pileal surface olivaceous-buff, slightly thick-walled, rarely branched, with simple septa, interwoven, 3–7 µm in diameter. Generative hyphae in context olivaceous-buff, thick-walled, occasionally branched, with simple septa, parallel, 2–6 µm in diameter. Generative hyphae in spines olivaceous-buff, thin-walled, branched, mostly with simple septa, occasionally with clamp connections, more or less parallel along the spines, 2–4 µm in diameter. Generative hyphae in stipe clay-buff, thick-walled rarely branched, bearing simple septa, interwoven in the outer layer, parallel along the stipe in the inner layer, 3–8 µm in diameter.

**Cystidia**—Cystidia and other sterile hyphal elements absent.

**Basidia**—Basidia clavate, bearing four sterigmata and a basal simple septum, 16–36 × 5–7 µm; sterigmata, 1–4 µm; basidioles similar to basidia in shape, but slightly smaller.

**Spores**—Basidiospores subglobose to globose, hyaline, thin-walled, echinulate, IKI–, CB–, 4–5 (–5.5) × (3.5–) 4–4.5 (–5) µm, L = 4.7 µm, W = 4.3 µm, Q = 1–1.25 (n = 90/3, without the ornamentation).

**Additional specimens (paratypes) examined**—China, Sichuan Province, Pingwu County, Bazi, on the ground of forest dominated by trees of *Quercus* sp., alt. 1190 m, 18 September 2020, Cui 18534 (BJFC 035394) & Cui 18540 (BJFC 035401).

**Ecological habits**—*P. griseofuscus* was found in forest dominated by trees of *Quercus* sp., under a humid monsoon-climate in the northern subtropical region.

**Key to species of *Phellodon* from China**1. Pileal surface colored straw ------------------------------------------------------- *P. stramineus*1. Pileal surface differently -------------------------------------------------------------------------- 22. Pileal surface blackish blue to dark grey ----------------------------------- *P. atroardesiacus*2. Pileal surface different colored ------------------------------------------------------------------ 33. Tissues change color in KOH -------------------------------------------------------------------- 43. Tissues unchanged in KOH ----------------------------------------------------- *P. subconfluens*4. Clamp connections exist -------------------------------------------------------------------------- 54. Clamp connections absent ------------------------------------------------------ *P. cinereofuscus*5. Clamp connections exist in spines -------------------------------------------------------------- 65. Clamp connections not exist in spines --------------------------------------------------------- 76. Spines brown after mature ------------------------------------------------------- *P. griseofuscus*6. Spines white after mature --------------------------------------------------------*P. perchocolatus*7. Pileal surface tomentose and azonate-----------------------------------------*P. crassipilieatus*7. Pileal surface glabrous and zonate----------------------------------------------*P. yunnanensis*

## 4. Discussion

In this study, phylogenetic analyses of *Phellodon* were conducted based on the ITS sequences and the combined ITS + nLSU + nSSU + RPB1 + RPB2 sequences to confirm the affinities of the new species and reveal the relationships of *Phellodon* species.

*Phellodon crassipileatus* formed a single lineage different from other species of *Phellodon* in our phylogenetic analyses (Figure 1 and Figure 2). Morphologically, *P. crassipileatus* is similar to *P. griseofuscus* in having infundibuliform and dark brown pileus. However, *P. griseofuscus* can be distinguished from *P. crassipileatus* by its fibrillose pileus, brown spines after maturity, presence of clamp connections in generative hyphae of spines, and longer basidia (22–55 × 5–6 µm).

*Phellodon griseofuscus* is closely related to *P. violascens* (Alb. and Schwein.) A.M. Ainsw. and *P. fuligineoalbus* (J.C. Schmidt) R.E. Baird in our phylogenetic analyses (Figure 1 and Figure 2). *Phellodon violascens* is similar to *P. griseofuscus* in having solitary or gregarious basidiomata and larger basidiospores measuring 4.5–5.4 × 4.3–4.5 µm [22]. However, *P. violascens* differs from *P. griseofuscus* by its white to flesh brown basidiomata and lack of clamp connections. *Phellodon fuligineoalbus* differs from *P. griseofuscus* by its yellow-white or light brown basidiomata, lack of clamp connections, and larger basidiospores measuring 4–6 × 4–5 µm [40]. *Phellodon yunnanensis* B.K. Cui and C.G. may be confused with *P. griseofuscus* in having white to brown spines. However, *P. yunnanensis* can be distinct from *P. griseofuscus* by its smaller basidiospores measuring 3.5–4.5 × 3–4 µm [28] and shorter clavate basidia measuring 24–27 × 6–7 µm [28].

*Phellodon perchocolatus* and *P. cinereofuscus* B.K. Cui and C.G. Song clustered together and then grouped with *P. mississippiensis* R.E. Baird, L.E. Wallace and G. Baker, forming a high supported lineage (98% MP, 100% ML, 1.00 BPP) in our phylogenetic trees (Figure 1 and Figure 2). Morphologically, *P. cinereofuscus* is similar to *P. perchocolatus* in having infundibuliform basidiomata and white pileal margin. However, *P. cinereofuscus* differs from *P. perchocolatus* by its reddish brown to cinnamon brown pileal surface, glabrous basidiomata and lack of clamp connections [28]. *Phellodon mississippiensis* is similar to *P. perchocolatus* in having solitary or gregarious basidiomata, and subglobose to globose basidiospores. However, *P. mississippiensis* can be distinguished from *P. perchocolatus* by its white, light orange to light brown pileal surface and shorter basidia measuring 16–22 × 5–6 µm [27]. Moreover, tissues in *P. mississippiensis* turned light to dark brown in KOH while turned to olive green in *P. perchocolatus*.

## 5. Conclusions

This study not only fills in the blank of multiple gene fragments of *Phellodon*, but also enriches the species diversity of the genus, which will promote the taxonomy and phylogeny of the genus. This is the first step to infer the phylogeny of *Phellodon* on the basis of multiple genes rather than ITS sequences. Therefore, this study provides a basis for further research on *Phellodon*. However, only a few species of *Phellodon* with available multiple genes could be used for the analyses, which limited the systematic study of the genus. For the time being, the best gene marker for the identification of *Phellodon* is ITS, while more samples with more gene markers including TEF, RPB1, and RPB2 are needed to further investigate the species diversity and phylogenetic relationships of *Phellodon* species.

## Figures and Tables

**Table 1:** A list of species, specimens and GenBank accession numbers of sequences used in this study.

Species	Specimen No.	Locality	GenBank Accession No.				
			ITS	nLSU	nSSU	RPB1	RPB2
*Amaurodon aquicoeruleus*	UK 452	Australia	AM490944	AM490944	-	-	-
*A. viridis*	TAA 149664	Russia	AM490942	AM490942	-	-	-
*Hydnellum atrospinosum*	Yuan 6520	China	MW579912	-	MW579912	-	-
*H. atrospinosum*	Yuan 6495	China	MW579938	MW579885	MW579911	-	-
*H. suaveolens*	ELarsson 139-09	Norway	MK602734	MK602734	-	-	-
*H. suaveolens*	ELarsson 8-14	Sweden	MK602735	MK602735	-	-	-
*Phellodon alboniger*	REB-70	USA	KC571749	-	-	-	-
*P. alboniger*	REB-57	USA	JN135206				
*P. atratus*	CL-72	Canada	MK281471	-	-	-	-
*P. atratus*	DAVFP 28189	Canada	HQ650766	-	-	-	-
*P. atroardesiacus*	Cui 18449	China	MZ221189	**MZ225598**	**MZ225636**	-	-
*P. atroardesiacus*	Cui 18457	China	MZ225577	**MZ225599**	**MZ225637**	-	-
*P. atroardesiacus*	Cui 18458	China	MZ225633	**MZ225600**	**MZ225638**	-	-
*P. atroardesiacus*	Cui 18459	China	MZ225634	**MZ225601**	**MZ225639**	-	-
*P. atroardesiacus*	Cui 16951	China	MZ225632	**MZ225597**	**MZ225635**	**MZ343209**	**MZ343197**
*P. brunneoolivaceus*	REB-166	USA	KC571752	-	-	-	-
*P. cinereofuscus*	Cui 14231	China	MZ225579	-	-	-	-
*P. cinereofuscus*	Cui 16940	Australia	MZ225580	**MZ225602**	**MZ225640**	**MZ343210**	**MZ343198**
*P. cinereofuscus*	Cui 16944	China	MZ225581	**MZ225603**	**MZ225641**	**MZ343211**	**MZ343199**
*P. cinereofuscus*	Cui 16945	China	MZ225582	**MZ225604**	**MZ225642**	-	-
*P. cinereofuscus*	Cui 16962	China	MZ225583	**MZ225605**	**MZ225643**	**MZ352084**	**MZ343200**
*P. cinereofuscus*	Cui 16963	China	MZ225584	**MZ225606**	**MZ225644**	**MZ352085**	**MZ343201**
*P. confluens*	WAT 28574	UK	EU622361	-	-	-	-
*P. confluens*	E00 186901	UK	EU622362	-	-	-	-
*P. crassipileatus*	Cui 18532	China	**OL449267**	**OL439037**	**OL439027**	-	-
*P. crassipileatus*	Cui 18533	China	**OL449268**	**OL439038**	**OL439028**	-	-
*P. ellisianus*	REB-264	USA	KC571757	-	-	-	-
*P. ellisianus*	REB-407	USA	KC571759	-	-	-	-
*P. fibulatus*	REB-168	USA	JN135205	-	-	-	-
*P. fibulatus*	REB-34	USA	KC571761	-	-	-	-
*P. fuligineoalbus*	REB-271	USA	KC571760	-	-	-	-
*P. fuligineoalbus*	REB-285	USA	JN135196	-	-	-	-
*P. fuligineoalbus*	SL8	-	EU622316	-	-	-	-
*P. griseofuscus*	Cui 18544	China	**OL449265**	**OL439035**	**OL439025**	**OL456229**	**OL449087**
*P. griseofuscus*	Cui 18561	China	**OL449266**	**OL439036**	**OL439026**	-	-
*P. melaleucus*	LH4	UK	EU622368	-	-	-	-
*P. melaleucus*	E00219373	UK	EU622369	-	-	-	-
*P. melaleucus*	Cui 18614	China	**OL449262**	**OL439032**	**OL439022**	**OL456228**	-
*P. melaleucus*	Cui 18620	China	**OL449263**	**OL439033**	**OL439023**	-	-
*P. melaleucus*	Cui 18623	China	**OL449264**	**OL439034**	**OL439024**	-	-
*P. mississippiensis*	MS-1	USA	JN247563	-	-	-	-
*P. mississippiensis*	MS-3	USA	JN247564	-	-	-	-
*P. niger*	REB-46	USA	JN135202	-	-	-	-
*P. niger*	REB-282	USA	KC571766	-	-	-	-
*P.* cf. *nothofagi*	MES-175	Chile	MH930224	-	-	-	-
*P. perchocolatus*	Cui 18534	China	**OL449259**	**OL439029**	**OL439020**	**OL456227**	-
*P. perchocolatus*	Cui 18536	China	**OL449260**	**OL439030**	-	-	-
*P. perchocolatus*	Cui 18540	China	**OL449261**	**OL439031**	**OL439021**	-	-
*P. putidus*	REB-8	USA	JN135200	-	-	-	-
*P. secretus*	0097	Russia	MG597404	-	-	-	-
*P. sinclairii*	PDD 89028	New Zealand	GU222291	-	-	-	-
*P. stramineus*	Cui 16942	China	MZ225585	**MZ225607**	**MZ225645**	**MZ352086**	-
*P. stramineus*	Cui 16943	China	MZ225586	**MZ225608**	**MZ225646**	**MZ352087**	**MZ343202**
*P. stramineus*	Cui 16956	China	MZ225587	**MZ225609**	**MZ225647**	**MZ352088**	**MZ343203**
*P. stramineus*	Cui 16959	China	MZ225588	**MZ225610**	**MZ225648**	**MZ352089**	**MZ343204**
*P. stramineus*	Cui 16961	China	MZ225589	**MZ225611**	**MZ225649**	**MZ352090**	**MZ343205**
*P. stramineus*	Cui 16964	China	MZ225590	**MZ225612**	**MZ225650**	**MZ352091**	-
*P. subconfluens*	Yuan 11123	China	MK677464	-	-	-	-
*P. subconfluens*	Yuan 11150	China	MK677465	-	-	-	-
*Phellodon* sp.1	REB-83	USA	KC571747	-	-	-	-
*Phellodon* sp.1	REB-325	USA	KC571748	-	-	-	-
*P. tomentosus*	SL70	UK	EU622381	-	-	-	-
*P. tomentosus*	LH22	UK	EU622382		-	-	-
*P. yunnanensis*	Cui 14292	China	MZ225591	-	-	-	-
*P. yunnanensis*	Cui 14294	China	MZ225592	-	-	-	-
*P. yunnanensis*	Cui 17097	China	MZ225593	**MZ225613**	**MZ225651**	-	**MZ343206**
*P. yunnanensis*	Cui 17129	China	MZ225594	**MZ225614**	**MZ225652**	-	**MZ343207**
*P. yunnanensis*	Cui 17131	China	MZ225595	**MZ225615**	**MZ225653**	-	**MZ343208**
*P. violascens*	2359-QFB-25626	Canada	KM406977	-	-	-	-
*Sarcodon imbricatus*	JRova 1408292	Sweden	MK602746	MK602746	-	-	-
*S. imbricatus*	ELarsson 384-10	Norway	MK602747	MK602747	-	-	-
*S. squamosus*	OF 177452	Norway	MK602768	MK602768	-	-	-
*S. squamosus*	OF 295554	Norway	MK602769	MK602769	-	-	-

New sequences are shown in bold.

## Data Availability

The data and results of this study are available upon reasonable request. Please contact the main author of this publication.
